# Dietary trends among Polish women in 2011–2022—cross-sectional study of food consumption frequency among women aged 20–50 in Silesia region, Poland

**DOI:** 10.3389/fnut.2023.1219704

**Published:** 2023-06-27

**Authors:** Agnieszka Białek-Dratwa, Teresa Kokot, Elżbieta Czech, Beata Całyniuk, Agata Kiciak, Wiktoria Staśkiewicz, Anita Stanjek-Cichoracka, Małgorzata Słoma-Krześlak, Olga Sobek, Maria Kujawińska, Martina Grot, Elżbieta Szczepańska, Małgorzata Muc-Wierzgoń

**Affiliations:** ^1^Department of Human Nutrition, Department of Dietetics, Faculty of Public Health in Bytom, Medical University of Silesia in Katowice, Katowice, Poland; ^2^Department of Preventive Medicine, Medical University of Silesia in Katowice, Bytom, Poland; ^3^Department of Epidemiology and Biostatistics, Faculty of Public Health in Bytom, Medical University of Silesia in Katowice, Katowice, Poland; ^4^Department of Technology and Food Quality Evaluation, Department of Dietetics, Faculty of Public Health in Bytom, Medical University of Silesia in Katowice, Katowice, Poland; ^5^Department of Biophysics, Faculty of Pharmaceutical Sciences in Sosnowiec, Medical University of Silesia in Katowice, Katowice, Poland; ^6^Laboratory of Transplant Immunology, Silesian Centre for Heart Diseases, Zabrze, Poland

**Keywords:** nutrition, women, diet, eating habits, lifestyle

## Abstract

**Background:**

Women’s nutrition should be different from that of men. Women have lower energy requirements than men. And the need for certain vitamins and minerals is higher in women, this applies to iron, calcium, magnesium, vitamin D and vitamin B9 (folic acid). This is related to hormonal changes including menstruation, pregnancy, breastfeeding and the onset of menopause. Through hormonal changes and the changing physiological state, women are at greater risk of anaemia, bone weakness and osteoporosis.

The aim of the study was to assess changes in the dietary pattern among women from the Silesian Agglomeration in Poland between 2011 and 2022.

**Material and method:**

The survey was conducted in 2011 (March–May 2011) and in 2022 (October–November 2022) among women living in the Silesian Agglomeration (Silesia region) in Poland aged 20–50. After consideration of the inclusion and exclusion criteria, 745 women were included in the final analysis, including 437 women screened in 2011 and 308 women screened in 2022.

The research tool used in this publication was a survey questionnaire consisting of 2 parts. The first part of the questionnaire consisted of demographic data. The second part of the study focused on the dietary habits of the women surveyed and the frequency of consumption of individual foods (FFQ).

**Results:**

More women in 2022 ate breakfast than in 2011 (77.6% vs. 63.8% *p* < 0.001), were more likely to eat breakfast I at home (73.1% vs. 62.5%; *p* < 0.001), were more likely to eat breakfast II (39.0% vs. 35.2%; *p* = 0.001), were more likely to eat breakfast II at home (28.6% vs. 19.2%; *p* = 0.002), and were more likely to eat lunch at work (16.6% vs. 3.4%; *p* < 0.001). Women in 2022 were more likely to consume fast-food (*p* = 0.001), salty snacks (chips, crisps) (*p* < 0.001) and sweets (*p* < 0.001). Women in 2022 were more likely to consume whole-grain bread (*p* < 0.001), wholemeal pasta (*p* < 0.001), brown rice (*p* < 0.001), oatmeal (*p* < 0.001), buckwheat groats (*p* = 0.06), and bran (*p* < 0.001) than women in 2011. They were less likely to consume white bread (*p* < 0.0001), light pasta (*p* = 0.004), white rice (*p* = 0.008) and cornflakes (*p* < 0.001) in 2022.

Women in 2022 were significantly more likely to consume vegetables (*p* < 0.001) than women in 2011.

**Conclusion:**

Eating habits in Silesia region women changed between 2011 and 2022. In 2022, women were more likely to choose cereal products considered health-promoting and rich in dietary fiber (including whole-grain bread, whole-grain pasta, oatmeal, bran) were more likely to consume vegetables, dry pulses and vegetarian dinners, and consumed less meat, cured meats, fish and dairy products. Consumption of fast-food, salty snacks (such as chips) and sweets increased.

## 1. Introduction

Among its initiatives aimed at reducing behavioral risk factors, the World Health Organization’s (WHO) Global Action Plan for the Prevention and Control of Noncommunicable Diseases includes strategies for addressing unhealthy dietary patterns; other components include physical inactivity, tobacco use, and harmful alcohol use (1). The World Health Organization advises balancing energy intake, cutting back on saturated and trans fats while boosting consumption of unsaturated fats, increasing fruit and vegetable consumption, and cutting back on sugar and sodium intake. Many of these dietary objectives are found naturally in regional diets, like the Mediterranean diet, or are included in evidence-based diets created to lower disease risk, like the Mediterranean-DASH intervention for neurodegenerative delay (MIND) and dietary approaches to stop hypertension (DASH) diets (2–4).

A balanced diet is one that delivers enough macronutrients to satisfy the body’s energy and physiologic needs without causing excess intake, as well as enough micronutrients and fluids to satisfy its physiological needs. Carbohydrates, proteins, and lipids, collectively known as macronutrients, provide the energy required for cellular functions essential for daily operation. Minimal amounts of micronutrients (vitamins and minerals) are necessary for healthy growth, development, metabolism, and physiologic function (5). Proteins in food offer both energy and amino acids. Animal and plant sources both contribute to dietary proteins, although the former is thought to be a richer source because of the diversity of amino acids, high digestibility, and higher bioavailability. Saturated fatty acids, which are present in animal-based sources of protein, have been associated with cancer, dyslipidemia, and cardiovascular disease. Although the exact processes are unclear, red meat, and processed meat in particular, has been associated to an increased risk of colorectal cancer (6–8).

Trans fats and, to a lesser extent, saturated fats are linked to adverse health impacts, including an increased risk of death, whereas unsaturated fats are linked to decreased cardiovascular and mortality risks. Due to their necessity for healthy growth and reproduction but inability to be created by the body, essential fatty acids, which include the omega-3 and omega-6 families of polyunsaturated fatty acids, must be consumed. Omega-3 fatty acids, particularly eicosapentaenoic acid (EPA) and docosahexaenoic acid (DHA), have been extensively researched for their potential health benefits, with evidence suggesting positive effects such as cardioprotection, preventing cognitive decline, reducing inflammation, sustaining muscle mass, and improving systemic insulin resistance. EPA and DHA are found in seafood, particularly oily fish, and supplements are widely available for those who cannot reach the prescribed intakes through diet alone. Nuts, certain seeds, and plant oils contain alpha-linolenic acid, the primary omega-3 fatty acid found in plants (9–12).

Cereals, fruits, legumes, and vegetables are the richest sources of carbohydrates, which are the body’s main energy source. Whole grains are preferred over processed grains in terms of their health advantages since processed grains have less fiber and micronutrient content because their germ and cereal have been removed during milling. A higher intake of whole grains has been associated with a lower risk of coronary heart disease, stroke, cardiovascular disease, and cancer, as well as a lower risk of death from any cause, cardiovascular disease, cancer, respiratory disease, diabetes, and infectious disease, according to meta-analyses of prospective cohort studies. Both calories and dietary fiber, which encourages satiety and has positive benefits on digestion, cholesterol levels, and glycemic management, are present in fresh fruits and vegetables. The benefits of consuming fibre in the daily diet are based on the type of fibre consumed: soluble (e.g., pectin, gums, plant mucilage, polysaccharides), or insoluble (e.g., cellulose, lignans). The main sources of insoluble fibre are cereal products, especially whole-grain cereals such as whole-grain bread, whole-grain pasta, bran, groats and whole-grain rice. Soluble fibre can be found in fruit and vegetables. Insoluble dietary fibre stimulates saliva secretion, increases the volume of food content resulting in a greater feeling of satiety, stimulates blood circulation and intestinal peristalsis, protects against constipation, reduces the risk of intestinal diverticulosis, rectal polyps and varices, and colon cancer. Soluble dietary fibre loosens faecal masses, thus preventing constipation, reduces cholesterol absorption, lowers the rate of carbohydrate absorption, thus slowing the rise in blood glucose after a meal. Studies have observed that there is a link between a low intake of dietary fibre and increased risk and mortality from cardiovascular disease (including ischaemic heart disease and cerebrovascular disease). Fresh fruits and vegetables are also significant sources of phytochemicals, which are bioactive substances believed to be responsible for many of the health advantages connected with fruit and vegetable consumption (13–19). Examples of phytochemicals include polyphenols, phytosterols, and carotenoids. These many phytochemicals’ confusing molecular effects include their antioxidative abilities, control over nuclear transcription factors, lipid metabolism, and production of inflammatory mediators. For instance, studies on flavonoids have revealed that these phytochemicals have some advantages in the treatment of obesity and diabetes by increasing insulin secretion and reducing insulin resistance. The risk of noncommunicable diseases (NCDs) such hypertension, cardiovascular disease, chronic obstructive pulmonary disease, lung cancer, and metabolic syndrome has been demonstrated to be inversely related to fruit and vegetable consumption (20–23). Nowadays, with the pace of life quickening and availability to processed foods being simpler than ever, it’s crucial to pay attention to what we consume and how it affects our bodies.

The main purpose of the study was to assess changes in the diet of women from the Silesian Agglomeration in Poland in the years 2011–2022. The main purpose of the study was based on the following specific purposes: a comparison was made between the frequency of consumption of particular food products, taking into account their health-promoting aspects (vegetables, fruit, dry leguminous seeds, cereal products with high content of dietary fibre, yoghurts, cottage cheese, fish) and those which may negatively affect health (fast food, sweets, salty snacks, fried food, cereal products from refined flour with low content of dietary fibre, products containing sugar, e.g., sweet cheeses among Polish women in 2011 and 2022 sweet cheeses) in 2011 and 2022 among Polish women. The study asked the following research questions:

- Have there been changes in dietary patterns among women between 2011 and 2022?
- Has there been an increase in the frequency of consumption of foods considered health-promoting among women?
- Has the frequency of consumption of products negatively affecting health decreased?

## 2. Materials and methods

### 2.1. Test sample

The survey was conducted in 2011 (March–May 2011) and in 2022 (October–November 2022) among women living in the Silesian Agglomeration (Silesia region) in Poland aged 20–50. The 2011 survey was conducted among random selected women living in the Silesia region. The survey in 2022, on the other hand, was conducted anonymously using the Computer-Assisted Web Interview (CAWI) method; the online survey was distributed in local forums and discussion groups among women residing in the Silesia region. The 2022 survey was conducted using the CAWI method; the sampling was completely random (according to the assumed inclusion and exclusion criteria of the survey). However, some risk of error should be taken into account that the female participants of the survey showed more interest in nutrition.

### 2.2. Inclusion and exclusion criteria

The inclusion criteria for the study were: female gender, residence in the Silesian Agglomeration, consent to participate, and correct and complete completion of the questionnaire.

On the other hand, the criteria for exclusion from the study were: gender other than female, age below 20 years and above 50 years, use of an unconventional diet (e.g., vegan, vegetarian, ketogenic, low-carb diet), disease entity determining elimination of the entire food group, place of residence other than Silesia region, lack of consent to participate in the study, incorrectly completed questionnaire.

After consideration of the inclusion and exclusion criteria, 745 women were included in the final analysis, including 437 women screened in 2011 and 308 women screened in 2022.

### 2.3. Research tool

The research tool used in this publication was a survey questionnaire consisting of 2 parts. The first part of the questionnaire consisted of demographic data. The study obtained the following demographic data: gender, age, education (primary, vocational, secondary, tertiary) and body weight and height—from this, body mass index (BMI) was calculated and interpreted based on World Health Organisation (WHO) recommendations for adults: <18.5 kg/m^2^—underweight, 18.5–24.9 kg/m^2^—average weight, 25.0–29.9 kg/m^2^—overweight, 30.0–34.9 kg/m^2^—grade I obesity, 35.0–39.9 kg/m^2^—grade II obesity, >40 kg/m^2^ grade III obesity (24). The levels of education adopted in the study were set according to Polish legal regulations: primary education, vocational education, secondary education (high school) and higher education (university).

The second part of the study focused on the dietary habits of the women surveyed and the frequency of consumption of individual foods (FFQ). The study assessed: the number of meals consumed per day, frequency of consumption of individual meals, place of consumption of meals, and the fact of eating meals with other family members. In the study of the frequency of consumption (FFQ) of individual food products, a 6-grade scale was taken into account: I do not consume at all, several times a month, several times a week, once a day, 2–3 times a day, 4 and more times a day to standardise the results. The consumption of fast-food products (including chips, casseroles, hamburgers, and pizza); salty snacks (chips, crisps, etc.); cereal products (divided by the number of meals consumed and the number of meals consumed); and other food products (divided by the number of meals consumed were taken into account); cereal products (subdivided into those that are health-promoting: wholemeal bread, wholemeal pasta, brown rice, oatmeal, buckwheat groats, muesli, bran and products with lower nutritional value: light bread—not made from wholemeal flour, pasta made from refined flour, white rice, cornflakes), vegetables, fruit, potatoes and dry pulses such as soya/lentils/peas/beans; dairy products and dairy products (yoghurt, buttermilk, kefir, white cheese, cottage cheese, sugar-sweetened fruit cheese, yellow cheese), meat and fish (meat, fish, cold cuts), vegetarian dinners. The study also included the consumption of sweets, drinks such as sugar-sweetened and unsweetened coffee and tea, sugar-sweetened and unsweetened juices, water, sugar-sweetened and unsweetened juices, water, sugar-sweetened and unsweetened carbonated drinks, and energy drinks. An assessment was also made of the thermal treatments most commonly used for meal preparation (boiling, steaming, frying, fat-free frying, baking, braising, grilling), as well as the type of fat used for frying and bread spreads (butter, lard, olive oil, vegetable oil, butter and margarine mix, hardened margarine).

The FFQ questionnaire was developed based on the principles of nutrition in force in 2011 for women in Poland. It was based on the principles of nutrition of the Polish Institute of Food and Nutrition in Warsaw (IŻŻ). In Poland in 2011, the institution responsible for setting nutrition standards was the IŻŻ (25, 26). The nutrition rules for women in 2022 have changed. They were developed by the National Centre for Nutrition Education (NCEZ), which is currently responsible for setting nutrition standards in Poland (27, 28). Professor Jarosz’s expert team established the nutrition standards in force in 2011(IŻŻ) and the standards in force in 2022 (NCEZ) despite the change in the name of the scientific unit.

### 2.4. Statistical analysis

The statistical analysis used descriptive and analytical methods available in Statistica v. 13.3 (StatSoft Inc., Tulsa, OK, United States). The statistical significance of differences between frequencies of qualitative variables was assessed based on the results of Pearson’s chi-square test and the test with Yates correction. Analysis of correlations was based on Kendall’s Tau coefficient and V-Cramer. The arithmetic mean (*M*) and standard deviation (SD), as well as the median (Me) and interquartile range (IQR) values were used to describe BMI, body weight, height and age of the study group. Statistical significance of differences between distributions of quantitative variables was assessed using the Mann–Whitney *U* test. Interpretation of the results was carried out based on the criterion of statistical significance *p* < 0.05.

### 2.5. Ethical consents

All study participants were informed of the purpose of the study, its anonymity and asked to accept the data sharing policy. Information about conscious and voluntary participation in the study was included at the beginning of the questionnaire. Due to the nature of the study, consent was sought from the Bioethics Committee of the Silesian Medical University in Katowice. In accordance with Resolution No. KNW/0022/KB1/151/I/11 of 06.12.2011. The Bioethics Committee took a positive opinion on the project of a medical experiment concerning the study of dietary habits in different groups of the population in terms of the occurrence of overweight and obesity.

## 3. Results

Table 1 shows the characteristics of the study group of women with respect to the study year 2011 and 2022. There were differences among the studied women in terms of age (*p* < 0.001), mean body weight (*p* < 0.001), mean height (*p* < 0.001), mean BMI (*p* = 0.02), place of residence (*p* < 0.001), BMI interpretation (*p* < 0.001) and education (*p* < 0.001). Considering the results, it should be noted that the mean BMI increased by 1.38 kg/m^2^.

**Table 1:** Characteristics of the study group of women by year of study 2011 and 2022.

Variable	2011		2022		Total 2011 and 2022		Test *p* correlation coefficient
	*n*	(%)	*n*	(%)	*N*	(%)	
Woman	437 (58.7%)		308 (41.3%)		745 (100.0%)		
Mean age (years) (median; interquartile range)	38.67 ± 4.74 (39.00; 7.00)		33,90 ± 6.70 (35.00; 8.50)		36.69 ± 6.10 (37.00; 7.00)		*U* = 10.00 *p* < 0.001
Mean body weight (kg) (median; interquartile range)	64.93 ± 11.83 (63.00; 13.00)		70.35 ± 16.97 (67.00; 19.50)6		67.17 ± 14.42 (64.00; 15.00)		*U* = −3.93 *p* < 0.001
Average height (cm) (median; interquartile range)	164.28 ± 5.97 (164.00; 8.00)		166.32 ± 5.99 (165.00; 8.00)		165.12 ± 6.06 (164.00; 10.00)		*U* = −4.05 *p* < 0,001
Mean BMI (kg/m^2^) (median; interquartile range)	24.01 ± 3.88 (23.42; 4.68)		25.39 ± 5.82 (24.09; 6.25)		24.58 ± 4.83 (23.59; 5.17)		*U* = −2.26 *p* = 0.024
Residence: city	434	(99.3%)	251	(81.5%)	685	(91.9%)	Chi^2^ = 77.48 *p* < 0.001 *τ*_b_ = −0.32
Village	3	(0.7%)	57	(18.5%)	60	(8.1%)	
BMI interpretation: underweight	9	(2.1%)	9	(2.9%)	18	(2.4%)	Chi^2^ = 17.6 *p* < 0.001 *τ*_c_ = 0.11
Normweight	286	(65.5%)	169	(54.9%)	455	(61.1%)	
Overweight	109	(24.9%)	78	(25.3%)	187	(25.1%)	
Obesity	33	(7.6%)	52	(16.9%)	85	(11.4%)	
Education: basic	36	(8.2%)	0	(0.0%)	36	(4.8%)	Chi^2^ = 222.45 *p* < 0.001 *τ*_c_ = 0.57
professional	107	(24.5%)	8	(2.6%)	115	(15.4%)	
Medium	178	(40.7%)	52	(16.9%)	230	(30.9%)	
Higher	116	(26.6%)	248	(80.5%)	364	(48.9%)	

Chi^2^, Pearson’s chi^2^ test; *U*, Mann–Whitney *U* test; *p*, significance level, correlation coefficient: *τ*_c_, Kendall’s *τ*_c_; *τ*_b_, Kendall’s *τ*_b_; *V*_C_, Cramer’s *V*.

Table 2 shows the results for the number of meals consumed by the female respondents and the place of consumption. Comparing meal intake by location and time, in 2011 women were more likely to eat three and four meals per day, while in 2022 some women extended the number of meals to five (18.5% vs. 24.7%; *p* = 0.29). More women in 2022 ate breakfast than in 2011 (77.6% vs. 63.8% *p* < 0.001), were more likely to eat breakfast I at home (73.1% vs. 62.5%; *p* < 0.001), were more likely to eat breakfast II (39.0% vs. 35.2%; *p* = 0.001), were more likely to eat breakfast II at home (28.6% vs. 19.2%; *p* = 0.002), and were more likely to eat lunch at work (16.6% vs. 3.4%; *p* < 0.001). A not great proportion of women in both 2011 and 2022 did not eat lunch at all.

**Table 2:** Meal consumption characteristics by location and time by 2011 and 2022.

Variable	2011		2022		Total 2011 and 2022		Test *p* correlation coefficient
	*n*	(%)	*n*	(%)	*N*	(%)	
How many meals do you eat in a day?							
1–2 meals	24	(5.5%)	13	(4.2%)	37	(5.0%)	Chi^2^ = 5.01 *p* = 0.287 *τ*_c_ = 0.08
3 meals	135	(30.9%)	82	(26.6%)	217	(29.1%)	
4 meals	181	(41.4%)	125	(40.6%)	306	(41.1%)	
5 meals	81	(18.5%)	76	(24.7%)	157	(21.1%)	
Over 5	16	(3.7%)	12	(3.9%)	28	(3.7%)	
Do you eat breakfast every day?							
Not	77	(17.6%)	16	(5.2%)	93	(12.5%)	Chi^2^ = 27.43 *p* < 0.001 *V*_C_ = 0.19
Not always	81	(18.6%)	53	(17.2%)	134	(18.0%)	
Yes	279	(63.8%)	239	(77.6%)	518	(69.5%)	
Where do you eat your first breakfast most often?							
I do not eat	31	(7.1%)	9	(2.9%)	40	(5.4%)	*Y* = 17.37 *p* < 0.001 *V*_C_ = 0.15
Home	273	(62.5%)	225	(73.1%)	498	(66.8%)	
On the way to work	2	(0.5%)	6	(1.9%)	8	(1.1%)	
At work	131	(29.9%)	68	(22.1%)	199	(26.7)	
Do you eat a second breakfast every day?							
Not	177	(40.5%)	87	(28.2%)	264	(35.4%)	Chi^2^ = 13.08 *p* = 0.001 *V*_C_ = 0.13
Not always	106	(24.3%)	101	(32.8%)	207	(27.8%)	
Yes	154	(35.2%)	120	(39.0%)	274	(36.8%)	
Where do you eat your second breakfast most often?							
I do not eat	124	(28.4%)	60	(19.5%)	184	(24.7%)	*Y* = 16.65 *p* = 0.002 *V*_C_ = 0.15
Home	84	(19.2%)	88	(28.6%)	172	(23.1%)	
On the way to work	4	(0.9%)	1	(0.3%)	5	(0.7%)	
Elsewhere	8	(1.8%)	12	(3.9%)	20	(2.7%)	
At work	217	(49.7%)	147	(47.7%)	364	(48.8%)	
Where do you eat lunch most often?							
I do not eat lunch	5	(1.1%)	2	(0.6%)	7	(0.9%)	*Y* = 39.07 *p* < 0.001 *V*_C_ = 0.23
Home	417	(95.4%)	255	(82.8%)	672	(90.2%)	
At work	15	(3.4%)	51	(16.6%)	66	(8.7%)	
How often do you eat dinner together with other family members							
Daily	160	(36.6%)	104	(33.8%)	264	(35.4%)	Chi^2^ = 5.79 *p* = 0.122 *τ*_c_ = −0.05
Several times a week	166	(38.0%)	114	(37.0%)	280	(37.6%)	
Once a week. Usually at the weekend	102	(23.3%)	74	(24.0%)	176	(23.6%)	
Less than once a week	9	(2.1%)	16	(5.2%)	25	(3.4%)	

Chi^2^, Pearson’s chi^2^ test; *Y*, chi^2^ test with Yates correction; *p*, significance level, correlation coefficient: *τ*_c_, Kendall’s *τ*_c_; *V*_C_, Cramer’s *V*.

Table 3 assesses the frequency of consumption of anti-health foods such as fast-food (casseroles, burgers, pizza, chips), salty snacks and sweets. Women in 2022 were more likely to consume fast-food (*p* = 0.001), salty snacks (chips, crisps) (*p* < 0.001) and sweets (*p* < 0.001).

**Table 3:** Characteristics of the consumption of anti-health products in the study group of women by year of study.

Variable	2011		2022		Total 2011 and 2022		Test *p* correlation coefficient
	*n*	(%)	*n*	(%)	*N*	(%)	
How often do you eat casseroles, hamburgers, pizza etc.?							
At all	114	(26.1%)	47	(15.3%)	161	(21.6%)	*Y* = 16.37 *p* = 0.001 *τ*_c_ = 0.12
Several times a month	315	(72.1%)	247	(80.2%)	562	(75.4%)	
Several times a week	7	(1.6%)	13	(4.2%)	20	(2.7%)	
Once a day	1	(0.2%)	1	(0.3%)	2	(0.3%)	
How often do you eat salty snacks (crisps, sticks)?							
At all	138	(31.6%)	54	(17.5%)	192	(25.8%)	*Y* = 51.84 *p* < 0.001 *τ*_c_ = 0.23
Several times a month	260	(59.5%)	176	(57.1%)	436	(58.5%)	
Several times a week	27	(6.2%)	68	(22.1%)	95	(12.8%)	
Once a day	10	(2.3%)	6	(2.0%)	16	(2.1%)	
2–3 times a day	2	(0.4%)	4	(1.3%)	6	(0.8%)	
How often do you eat sweets?							
At all	37	(8.5%)	26	(8.4%)	63	(8.5%)	*Y* = 25.65 *p* < 0.001 *τ*_c_ = 0.17
Several times a month	171	(39.1%)	78	(25.3%)	249	(33.4%)	
Several times a week	134	(30.7%)	94	(30.5%)	228	(30.6%)	
Once a day	69	(15.8%)	71	(23.1%)	140	(18.8%)	
2–3 times a day	23	(5.2%)	37	(12.0%)	60	(8.0%)	
4 or more times a day	3	(0.7%)	2	(0.7%)	5	(0.7%)	

*Y*, chi^2^ test with Yates correction; *p*, significance level; *τ*_c_, Kendall’s *τ*_c_ correlation coefficient.

Tables 4, 5 assess the consumption of cereal products. Women in 2022 were more likely to consume whole-grain bread (*p* < 0.001), wholemeal pasta (*p* < 0.001), brown rice (*p* < 0.001), oatmeal (*p* < 0.001), buckwheat groats (*p* = 0.06), and bran (*p* < 0.001) than women in 2011. They were less likely to consume white bread (*p* < 0.0001), light pasta (*p* = 0.004), white rice (*p* = 0.008) and cornflakes (*p* < 0.001) in 2022.

**Table 4:** Frequency of consumption of health-promoting cereal products among female respondents by year of study.

Variable	2011 *r*.		2022 *r*.		Total 2011 and 2022		Test *p* correlation coefficient
	*n*	(%)	*n*	(%)	*N*	(%)	
Wholemeal bread							
4 or more times a day	2	(0.5%)	0	(0.0%)	2	(0.3%)	*Y* = 32.27 *p* < 0.001 *τ*_c_ = 0.09
2–3 times a day	32	(7.3%)	20	(6.5%)	52	(7.0%)	
Once a day	84	(19.2%)	50	(16.2%)	134	(18.0%)	
Several times a week	90	(20.6%)	116	(37.7%)	206	(27.6%)	
Several times a month	143	(32.7%)	89	(28.9%)	232	(31.1%)	
at all	86	(19.7%)	33	(10.7%)	119	(16.0%)	
Wholemeal pasta							
Several times a week	15	(3.4%)	36	(11.7%)	51	(6.8%)	Chi^2^ = 168.10 *p* < 0.001 *τ*_c_ = 0.44
Several times a month	55	(12.6%)	155	(50.3%)	210	(28.2%)	
At all	367	(84.0%)	117	(38.0%)	484	(65.0%)	
Brown rice							
Several times a week	6	(1.4%)	15	(4.9%)	21	(2.8%)	*Y* = 81.21 *p* < 0.001 *τ*_c_ = 0.26
Once a day	7	(1.6%)	0	(0.0%)	7	(0.9%)	
Several times a month	63	(14.4%)	123	(39.9%)	186	(25.0%)	
At all	361	(82.6%)	170	(55.2%)	531	(71.3%)	
Oatmeal							
2–3 times a day	5	(1.1%)	5	(1.6%)	10	(1.3%)	*Y* = 97.34 *p* < 0.001 *τ*_c_ = 0.37
Once a day	19	(4.4%)	35	(11.4%)	54	(7.3%)	
Several times a week	32	(7.3%)	61	(19.8%)	93	(12.5%)	
Several times a month	117	(26.8%)	128	(41.6%)	245	(32.9%)	
At all	264	(60.4%)	79	(25.6%)	343	(46.0%)	
Buckwheat groats							
Once a day	3	(0.7%)	3	(1.0%)	6	(0.8%)	*Y* = 7.53 *p* = 0.056 *τ*_c_ = 0.08
Several times a week	14	(3.2%)	22	(7.1%)	36	(4.8%)	
Several times a month	240	(54.9%)	174	(56.5%)	414	(55.6%)	
At all	180	(41.2%)	109	(35.4%)	289	(38.8%)	
Muesli							
2–3 times a day	5	(1.1%)	1	(0.3%)	6	(0.8%)	*Y* = 6.35 *p* = 0.174 *τ*_c_ = −0.04
Once a day	23	(5.3%)	7	(2.3%)	30	(4.0%)	
Several times a week	52	(11.9%)	39	(12.7%)	91	(12.2%)	
Several times a month	128	(29.3%)	91	(29.5%)	219	(29.4%)	
At all	229	(52.4%)	170	(55.2%)	399	(53.6%)	
Bran							
2–3 times a day	1	(0.2%)	3	(1.0%)	4	(0.5%)	*Y* = 27.79 *p* < 0.001 *τ*_c_ = 0.07
Once a day	26	(6.0%)	4	(1.3%)	30	(4.0%)	
Several times a week	24	(5.5%)	25	(8.1%)	49	(6.6%)	
Several times a month	63	(14.4%)	77	(25.0%)	140	(18.8%)	
At all	323	(73.9%)	199	(64.6%)	522	(70.1%)	

Chi^2^, Pearson’s chi^2^ test; *Y*, chi^2^ test with Yates correction; *p*, significance level, correlation coefficient: *τ*_c_, Kendall’s *τ*_c_.

**Table 5:** Frequency of intake of nutrient-reduced cereal products among female respondents by year of study.

Variable	2011 *r*.		2022 *r*.		Total 2011 and 2022		Test *p* correlation coefficient
	*n*	(%)	*n*	(%)	*N*	(%)	
White bread							
4 or more times a day	8	(1.8%)	1	(0.3%)	9	(1.2%)	*Y* = 74.71 *p* < 0.001 *τ*_c_ = −0.31
2–3 times a day	148	(33.9%)	33	(10.7%)	181	(24.3%)	
Once a day	94	(21.5%)	58	(18.8%)	152	(20.4%)	
Several times a week	82	(18.8%)	90	(29.3%)	172	(23.1%)	
Several times a month	64	(14.6%)	85	(27.6%)	149	(20.0%)	
At all	41	(9.4%)	41	(13.3%)	82	(11.0%)	
Pasta							
Once a day	6	(1.4%)	0	(0.0%)	6	(0.8%)	*Y* = 13.50 *p* = 0.004 *τ*_c_ = −0.10
Several times a week	107	(24.5%)	57	(18.5%)	164	(22.0%)	
Several times a month	288	(65.9%)	211	(68.5%)	499	(67.0%)	
At all	36	(8.2%)	40	(13.0%)	76	(10.2%)	
White rice							
Once a day	5	(1.1%)	1	(0.3%)	6	(0.8)	*Y* = 11.90 *p* = 0.008 *τ*_c_ = −0.07
Several times a week	43	(9.8%)	33	(10.7%)	76	(10.2%)	
Several times a month	348	(79.6%)	221	(71.8%)	569	(76.4%)	
At all	41	(9.4%)	53	(17.2%)	94	(12.6%)	
Cornflakes							
2–3 times a day	7	(1.6%)	1	(0.3%)	8	(1.1%)	*Y* = 39.46 *p* < 0.001 *τ*_c_ = −0.22
Once a day	13	(3.0%)	2	(0.7%)	15	(2.0%)	
Several times a week	48	(11.0%)	13	(4.2%)	61	(8.2%)	
Several times a month	146	(33.4%)	71	(23.1%)	217	(29.1%)	
At all	223	(51.0%)	221	(71.7%)	444	(59.6%)	

*Y*, chi^2^ test with Yates correction; *p*, significance level, correlation coefficient: *τ*_c_, Kendall’s *τ*_c_.

The study also assessed the consumption of health-promoting foods such as vegetables, fruit, dry pulses and potatoes. Women in 2022 were significantly more likely to consume vegetables (*p* < 0.001) than women in 2011. More than half of women consumed vegetables 2–3 times a day (2022 48.4% vs. 2011 14.4%; *p* < 0.001). They were more likely to consume fruit 2–3 times a day in 2022 than 2011 (*p* = 0.118), but this difference is not statistically significant. They were also more likely to consume dry pulses such as soya, lentils, chickpeas, broad beans or beans (*p* < 0.001). In contrast, women were less likely to consume potatoes in 2022 than in 2011. (*p* < 0.001; Table 6).

**Table 6:** Frequency of vegetable, fruit, and legume consumption among female respondents by year of study.

Variable	2011 *r*.		2022 *r*.		Total 2011 and 2022		Test *p* correlation coefficient
	*n*	(%)	*n*	(%)	*N*	(%)	
Vegetables							
4 or more times a day	11	(2.5%)	20	(6.5%)	31	(4.1%)	*Y* = 128.99 *p* < 0.001 *τ*_c_ = 0.34
2–3 times a day	63	(14.4%)	149	(48.4%)	212	(28.5%)	
Once a day	162	(37.1%)	55	(17.8%)	217	(29.1%)	
Several times a week	157	(35.9%)	61	(19.8%)	218	(29.3%)	
Several times a month	36	(8.2%)	23	(7.5%)	59	(7.9%)	
At all	8	(1.8%)	0	(0.0%)	8	(1.1%)	
Potatoes							
2–3 times a day	12	(2.7%)	0	(0.0%)	12	(1.6%)	*Y* = 175.24 *p* < 0.001 *τ*_c_ = −0.44
Once a day	127	(29.1%)	14	(4.5%)	141	(18.9%)	
Several times a week	203	(46.4%)	109	(35.4%)	312	(41.9%)	
Several times a month	71	(16.3%)	173	(56.2%)	244	(32.8%)	
At all	24	(5.5%)	12	(3.9%)	36	(4.8%)	
Legumins							
2–3 times a day	7	(1.6%)	7	(2.3%)	14	(1.9%)	*Y* = 21.57 *p* < 0.001 *τ*_c_ = 0.17
Once a day	7	(1.6%)	10	(3.2%)	17	(2.3%)	
Several times a week	30	(6.9%)	43	(14.0%)	73	(9.7%)	
Several times a month	229	(52.4%)	172	(55.8%)	401	(53.8%)	
At all	164	(37.5%)	76	(24.7%)	240	(32.2%)	
Fruit							
4 or more times a day	8	(1.8%)	5	(1.6%)	13	(1.7%)	*Y* = 8.78 *p* = 0.118 *τ*_c_ = 0.07
2–3 times a day	80	(18.3%)	77	(25.0%)	157	(21.1%)	
Once a day	167	(38.2%)	116	(37.7%)	283	(38.0%)	
Several times a week	129	(29.5%)	71	(23.1%)	200	(26.9%)	
Several times a month	40	(9.2%)	34	(11.0%)	74	(9.9%)	
At all	13	(3.0%)	5	(1.6%)	18	(2.4%)	

*Y*, Chi^2^ test with Yates correction; *p*, significance level, correlation coefficient: *τ*_c_, Kendall’s *τ*_c_. The consumption of dairy products was assessed. Women in 2022 were less likely to consume natural yoghurt (*p* < 0.001), buttermilk (*p* < 0.001), natural cottage cheese (*p* < 0.001), yoghurt and flavoured cheese, e.g., fruit (*p* < 0.001), and yellow cheese (*p* = 0.005) than women in 2011. (Table 7).

The consumption of dairy products was assessed. Women in 2022 were less likely to consume natural yoghurt (*p* < 0.001), buttermilk (*p* < 0.001), natural cottage cheese (*p* < 0.001), yoghurt and flavoured cheese, e.g., fruit (*p* < 0.001), and yellow cheese (*p* = 0.005) than women in 2011 (Table 7).

**Table 7:** Frequency of consumption of dairy products such as natural yoghurts, cheeses, cottage cheese, and flavoured yoghurts and cheeses among female respondents by year of survey.

Variable	2011		2022		Total 2011 and 2022		Test *p* correlation coefficient
	*n*	(%)	*n*	(%)	*N*	(%)	
Natural yoghurt							
2–3 times a day	31	(7.1%)	6	(2.0%)	37	(5.0%)	Chi^2^ = 28.77 *p* < 0.001 *τ*_c_ = −0.20
Once a day	111	(25.4%)	45	(14.6%)	156	(20.9%)	
Several times a week	152	(34.8%)	114	(37.0%)	266	(35.7%)	
Several times a month	103	(23.6%)	103	(33.4%)	206	(27.7%)	
At all	40	(9.1%)	40	(13.0%)	80	(10.7%)	
Buttermilk							
2–3 times a day	14	(3.2%)	0	(0.0%)	14	(1.9%)	*Y* = 89.23 *p* < 0.001 *τ*_c_ = −0.34
Once a day	38	(8.7%)	5	(1.6%)	43	(5.8%)	
Several times a week	116	(26.5%)	31	(10.1%)	147	(19.7%)	
Several times a month	184	(42.1%)	148	(48.0%)	332	(44.6%)	
At all	85	(19.5%)	124	(40.3%)	209	(28.0%)	
Curd cheeses/ cottage cheese							*Y* = 22.52 *p* < 0.001 *τ*_c_ = −0.17
2–3 times a day	16	(3.7%)	3	(1.0%)	19	(2.6%)	
Once a day	37	(8.5%)	19	(6.2%)	56	(7.5%)	
Several times a week	195	(44.6%)	105	(34.1%)	300	(40.3%)	
Several times a month	163	(37.3%)	147	(47.7%)	310	(41.6%)	
At all	26	(5.9%)	34	(11.0%)	60	(8.0%)	
Fruit cheeses/yoghurts							
2–3 times a day	13	(3.0%)	1	(0.3%)	14	(1.9%)	*Y* = 173.19 *p* < 0.001 *τ*_c_ = −0.50
Once a day	34	(7.8%)	4	(1.3%)	38	(5.1%)	
Several times a week	154	(35.2%)	31	(10.1%)	185	(24.8%)	
Several times a month	148	(33.9%)	75	(24.3%)	223	(29.9%)	
At all	88	(20.1%)	197	(64.0%)	285	(38.3%)	
Yellow cheeses							
4 or more times a day	5	(1.1%)	1	(0.3%)	6	(0.8%)	*Y* = 16.73 *p* = 0.005 *τ*_c_ = −0.12
2–3 times a day	18	(4.1%)	11	(3.6%)	29	(3.9%)	
Once a day	65	(14.9%)	45	(14.6%)	110	(14.8%)	
Several times a week	231	(52.9%)	127	(41.2%)	358	(48.0%)	
Several times a month	98	(22.4%)	101	(32.8%)	199	(26.7%)	
At all	20	(4.6%)	23	(7.5%)	43	(5.8%)	

Chi^2^, Pearson’s Chi^2^ test; *Y*, Chi^2^ test with Yates correction; *p*, significance level, correlation coefficient: *τ*_c_, Kendall’s *τ*_c_.

In 2022, women in the study population consumed less meat than in 2011 (*p* < 0.001), consumption of cold cuts (*p* < 0.001) as well as fish decreased (*p* = 0.001). On the other hand, the consumption of vegetarian so-called meatless dinners increased (*p* < 0.001; Table 8).

**Table 8:** Frequency of intake of products that are a source of protein among female respondents by year of study.

Variable	2011		2022		Total 2011 and 2022		Test *p* correlation coefficient
	*n*	(%)	*n*	(%)	*N*	(%)	
Meat							
2–3 times a day	8	(1.8%)	10	(3.3%)	18	(2.4%)	*Y* = 23.93 *p* < 0.001 *τ*_c_ = −0.10
Once a day	69	(15.8%)	34	(11.0%)	103	(13.8%)	
Several times a week	278	(63.6%)	179	(58.1%)	457	(61.4%)	
Several times a month	75	(17.2%)	60	(19.5%)	135	(18.1%)	
At all	7	(1.6%)	25	(8.1%)	32	(4.3%)	
Fish							
2–3 times a day	3	(0.7%)	2	(0.6%)	5	(0.7%)	*Y* = 18.00 *p* = 0.001 *τ*_c_ = −0.11
Once a day	6	(1.4%)	6	(2.0%)	12	(1.6%)	
Several times a week	94	(21.5%)	45	(14.6%)	139	(18.7%)	
Several times a month	320	(73.2%)	225	(73.1%)	545	(73.1%)	
At all	14	(3.2%)	30	(9.7%)	44	(5.9%)	
Cold cuts							
4 or more times a day	8	(1.8%)	1	(0.3%)	9	(1.2%)	*Y* = 186.13 *p* < 0.001 *τ*_c_ = −0.50
2–3 times a day	46	(10.5%)	1	(0.3%)	47	(6.3%)	
Once a day	165	(37.8%)	25	(8.1%)	190	(25.5%)	
Several times a week	163	(37.3%)	152	(49.4%)	315	(42.3%)	
Several times a month	48	(11.0%)	117	(38.0%)	165	(22.2%)	
At all	7	(1.6%)	12	(3.9%)	19	(2.5%)	
Vegetarian lunches/dinners							
2 times a day	3	(0.7%)	13	(4.2%)	16	(2.2%)	*Y* = 162.14 *p* < 0.001 *τ*_c_ = 0.33
Once a day	3	(0.7%)	48	(15.6%)	51	(6.8%)	
Several times a week	106	(24.2%)	131	(42.5%)	237	(31.8%)	
Several times a month	267	(61.1%)	68	(22.1%)	335	(45.0%)	
At all	58	(13.3%)	48	(15.6%)	106	(14.2%)	

*Y*, chi^2^ test with Yates correction; *p*, significance level, correlation coefficient: *τ*_c_, Kendall’s *τ*_c_.

## 4. Discussion

Diet is a factor that significantly determines nutritional status. Many factors influence the health of any woman. The first is a lifestyle, which determines a person’s health to the greatest extent. Lifestyle components can include diet, physical activity, alcohol consumption or smoking. These components are modifiable factors and can be changed at any time, and every woman has the right to decide about her life. In addition, health is affected by the physical environment (soil, air and water pollution), the social environment (family situation, economic situation, housing or psychological problems), work (toxic substances, dust, noise), genetic conditions or the state of health services (29).

It has been modified over the last few years. One of the factors influencing changes in the population’s nutritional status is the lifestyle change caused by the COVID-19 pandemic. Restriction of leaving the residence, travel, remote work, social isolation, restriction of stationary shopping, and closure of gastronomy, gyms and other sports facilities required lifestyle adaptation to the restrictions in place (30). Studies analysing changes in the nutritional status of women of the Polish population during the COVID-19 pandemic indicate an increase in body weight and BMI. In a study by Blaszczyk-Bęben et al. (31). The body weight of 45.86% of the women studied during social isolation increased significantly. In contrast, Sidor and Rzymski (32) found that dietary changes during quarantine could potentially cause weight changes as a result of less physical activity, changes in food intake and stress associated with the new situation; 29.9% of respondents reported an increase in weight during quarantine and the maximum reported gain was 10 kg. Significantly higher gains were observed in overweight and obese subjects (32). Analogous data were obtained by Dobrowolski and Włodarek (33). The average weight gain during the first quarantine during the pandemic averaged 2 kg and was associated with decreased physical activity and increased consumption of total food and energy-dense products (33). The COVID-19 pandemic is not the only factor contributing to increased body weight and BMI over the past decade. The prevalence of obesity in adults in the United States aged 20 years and older, defined as a BMI ≥30 kg/m^2^, was 30.5% in 2000 and 39.8% 15 years later (34). The prevalence of severe obesity (BMI > 40) increased at an even more alarming rate in adults, moving from 3.9% in 2000 to 6.6% in 2010 (35). Changes in the global food system and sedentary lifestyles are widely recognised as the main reasons for the increase in obesity prevalence worldwide. In line with these trends, the main driver of the obesity epidemic appears to be Western lifestyle modernisation (36). Between 1997 and 2017, the prevalence of overweight increased in Polish women from 30% to 32.2%, while at the same time, the prevalence of obesity decreased from 19 to 16.1% (37). Stoś et al. (38) conducted the study in 2019/20 and included 900 women. The mean age was 51.7 ± 19.8 years. According to the WHO classification, 33.9% of women aged 18–29 were overweight or obese; the percentage of women with abnormal body weight increased with age. The highest percentage of women with abnormal body weight was aged 60–69 years (71.5% of women). Almost half of the women in the study (49.2%) had abnormal body weight, defined as overweight or obese (38).

Considering our results, it should be noted that the average body mass index BMI increased by 1.37 kg/m2. The % of women with obesity also increased from 7.6% in 2011 to 16.9% in 2022, and the number of overweight women was 24.9% in 2011 and 25.3% in 2022. The data presented confirms that overweight and obesity in the Polish female population was already a serious problem before the COVID-19 pandemic, the pandemic itself and the associated restrictions potentially exacerbating the issues raised in the study.

The changes we identified in dietary patterns among working-age women over 2011–2022 was significant. When embarking on the 2011 survey, we did not define assumptions in the questionnaire such as lockdown, total remote working, hybrid remote working, and widespread quarantines during which entire families were not allowed to leave their homes and flats for weeks at a time. The survey conducted in 2022 already took into account the new state of functioning in society associated with the COVID pandemic, which had prevailed for 2 years. At the same time, the survey was already conducted after solid pandemic restrictions, where the use of grocery shops, restaurants and fast-food bars was restored from before the pandemic period.

Research conducted during the COVID-19 pandemic showed that not everyone adapted to the new conditions to the same extent. This was also observed in the dietary habits of Polish adults over 40 years of age, those living with children, those living in regions with a higher gross domestic product and those who did not eat at home before the pandemic (39). Adherence to dietary recommendations was also influenced by psychological factors and the ability to cope with difficult situations and adapt quickly to changing circumstances (40). Many studies have addressed lifestyle characteristics in the last 3 years, including dietary adherence considering the COVID pandemic-19. The methodology of most studies was based on qualitative methods assessing dietary habits, taking into account the frequency of consumption of product groups and the direction of change in their consumption during the pandemic (41, 42). Studies highlight that the consequences of social isolation caused by COVID-19 can include a change in eating behaviour. Among others, the following were observed: increased breakfast intake, reduced consumption of fried foods and meals consumed in restaurants and fast food, and increased consumption of fresh produce (43–46). The frequency of consumption of fruit, legumes and vegetables also increased (47).

The study by Górecka et al. confirmed our obtained results regarding the frequency of consumption of specific product groups. Two opposing dietary patterns were observed: a pro-healthy one associated with increased consumption of whole-grain products, vegetables, fruit and water, and an unhealthy one with the consumption of processed meat, fast food, confectionery and alcohol and sweets was increased (39). In a study by Bolesławska et al. (48), a significantly higher percentage of women also declared eating five meals compared to the previous period (*p* < 0.0001), an increase in the frequency of consumption of potatoes excluding chips and chips (*p* = 0.0026), sweets (*p* = 0.0127), eggs (*p* = 0.0011) and canned meat (*p* = 0.0166), and a decrease in the consumption of fast food (*p* = 0.0038) and instant and ready-made soups (*p* = 0.0327). Vegetables among the women surveyed were consumed several times a day by 36.5% of respondents before the lockdown and 34.5% during the lockdown (*p* = 0.6747). The fruit was less popular (20.5% before lockdown vs. 23.5% during lockdown, *p* = 0.9220).

The changes in the women’s diets in the self-study included the distribution of the number of meals. In 2011, women were more likely to eat three or four meals daily; in 2022, some women expanded to five per day. More women in 2022 ate breakfast than in 2011 (77.6% vs. 63.8%) (*p* < 0.001), were more likely to eat breakfast at home (73.1% vs. 62.5%) (*p* < 0.001), were more likely to eat breakfast II (39.0% vs. 35.2%) (*p* = 0.001), more often had their second breakfast at home (28.6% vs. 19.2%) (*p* = 0.002), more often had their lunch at work (16.6% vs. 3.4%) (*p* < 0.001). The increase in the number of meals consumed is confirmed by a study by Bolesławska et al. (48). Also, in Oleszko’s 2019 study, most Silesia region women eat 4–5 meals (59.20%) (49).

A small percentage of women in 2011 and 2022 did not eat lunch. It was 1.14% in 2011 and 0.65% in 2022. In Poland, there is a traditional model of meal consumption that includes: breakfast eaten in the morning at home or after coming to work, followed by a second breakfast usually eaten at school/workplace/work around 10 a.m. to 12 p.m. The next meal is a traditional lunch eaten after returning from work, while in the evening, dinner is eaten, which is often served in the form of sandwiches. In Poland, a lunch-type meal at the workplace is still rare. Poles, irrespective of gender, are more likely to eat lunch at home after coming from work around 3 p.m., between 6 a.m. and 2 p.m., around 4 p.m. when they work between 7 a.m. and 3 p.m., and after 5 p.m. when they work until 4 p.m. According to Art, this may be due to Polish labour law, which only allows for one break during 8 h of work. 134 of the Labour Code, “an employee has the right to a break of at least 15 min counted as working time” (50), the lack of tradition of eating lunch at work, as well as the lack of places to eat meals at the workplace and financial issues (meals in the form of sandwiches prepared at home are cheaper than buying a ready-made lunch).

In their study, women in 2022 were more likely to consume fast food (*p* = 0.001), salty snacks (chips, crisps) (*p* < 0.001) and sweets (*p* < 0.001) than women in 2011. Similar results were obtained in a study by Sidor and Rzymski; one-third of the subjects admitted to consuming sweets daily during the lockdown period (32). In the 2019 study by Oleszko et al, the frequency of sweet consumption was lower than in their study 24.48% of women consumed sweets daily. In their study, more than 35.0% consumed sweets daily (49). Comparing fast-food consumption, the results from Oleszko et al. (49) are comparable to 9.2% consuming fast-food several times a week vs. their own study’s 9.41%.

Many vegetable compounds positively affect the cardiovascular system, prevent diabetes and cancer and reduce anxiety and depressive symptoms. This is due to phytonutrients found in plant foods or naturally occurring in plant foods (51). With advances in science, various chemical compounds in vegetables have been identified to have therapeutic effects. Among these, the most potent are antioxidants and bioflavonoids, the primary pigments in vegetables (52). Phytonutrients are believed to reduce the danger of many chronic diseases by defending against free radicals, detoxifying carcinogens and modifying metabolic activation, or influencing processes that modify the course of cancer cell development. In the daily diet, colourful vegetables are strongly associated with improved eye health, gastrointestinal health and reduced risk of cardiovascular disease, chronic disease and cancer (53).

According to Eurostat data, in Poland in 2014 0 portions of fruit and vegetables were consumed by 33.2.% of Polish women, 59.5% consumed 1–4 portions of fruit and vegetables per day, and 11.8% had 5 or more portions. In 2019 5 portions of fruit and vegetables were consumed by less than 10.6% of Polish women. 57.3% consumed 1–4 portions and 0 portions, as many as 32.2% of Polish women (54). Considering current Polish dietary recommendations, an average adult Polish woman should consume at least 400 g of vegetables and fruit per day—with a higher intake of vegetables than of fruit. Data from the Central Statistical Office in Poland indicate that the average monthly intake in 2020 was 11.98 kg excluding potatoes which gives a daily vegetable intake of 399 g. Compared to 2016, the average intake decreased by 21 g/day (55).

In our study, women in 2022 were significantly more likely to consume vegetables (*p* < 0.001) than women in 2011. More than half of the women consumed vegetables several times daily (2022 54.9% vs. 2011 16.9%). They were more likely to consume fruit several times a day in 2022 than in 2011. They were also more likely to consume dry pulses such as soya, lentils, chickpeas, broad beans or beans (*p* < 0.001). In contrast, women were less likely to consume potatoes in 2022 than in 2011 (*p* < 0.001). In the 2019 study by Oleszko et al., the largest number of women surveyed consumed 1–2 portions of fruit and vegetables daily (59.38%), and 32.12% declared 3–4 portions per day (49).

In the 2022 self-study, women were more likely to consume whole-grain bread (*p* < 0.001), wholemeal pasta (p < 0.001), brown rice (*p* < 0.001), oatmeal (*p* < 0.001), buckwheat groats (*p* = 0.056), and bran (*p* < 0.001) than women in 2011. In contrast, they were less likely to consume white bread (*p* < 0.001), pasta (*p* > 0.004), white rice (*p* = 0.008) and cornflakes (p < 0.001) in 2022. The more frequent choice of whole-grain bread than white bread (46.70% vs. 41.85%) confirms the study by Oleszko et al. (39).

In the 2019 study by Oleszko et al, the majority of women consumed milk and dairy products occasionally (46.00%) (39), confirming the trend we also observed in our study that women in 2022 were less likely to consume natural yoghurt (*p* < 0.001), buttermilk (*p* < 0.001), natural cottage cheese (*p* < 0.001), yoghurt and flavoured cheese, e.g., fruit (p < 0.001), and yellow cheese (*p* = 0.005 than women in 2011). The study by Oleszko et al. (39) also considered the consumption of fish, meat, and cold cuts—most respondents consumed fish several times a month (79.51%). Moreover, meat and cold cuts, 48.26% consumed several times a week. In the 2022 self-reported survey, women consumed meat less frequently than in 2011 (*p* < 0.001), consumption of cold cuts (*p* < 0.001), as well as fish, decreased (*p* = 0.001).

All these changes can be attributed to several factors: increased consumer and health awareness (increased consumption of vegetables, whole-grain cereals, choice of vegan dinners, reduced consumption of cold cuts and meat), diagnosis of food allergies and intolerances, especially when it comes to the consumption of dairy products and the widespread availability of alternative dairy products such as plant-based drinks, plant-based yoghurts based on soya, coconut, cashew nuts or almonds, dietary fashions (vegan dinners, alternative foods, e.g., plant-based yoghurts) and the price of products (especially fish).

### 4.1. Strengths and limitations

There are limitations to survey that need to be taken into account when evaluating and interpreting the results. The 2022 survey was based on an anonymous online questionnaire. The questionnaire was conducted using the CAWI method. This type of data collection is widely accepted and convenient for collecting large amounts of information in groups that are often difficult to reach. However, there is an inability to verify the data. The researchers in both 2011 and 2022 did not measure respondents’ weight and height, but these were declared by the respondents. Therefore, the weight and height of the respondents, and the BMI calculated from them, should be regarded more as a rough estimate than an exact value. The study was conducted in Silesia (Poland), where in other regions of Poland women’s diets may be different from those in our study, there is a limitation of the local dietary pattern, characteristic of this region of Poland. The studies conducted in both 2011 and 2021 cover the same population of women from the Silesian agglomeration, so it is reasonable to compare this population. This study uses a simplified approach to summarise the overall prevalence of food group intake by relating it to the recommendations of the Institute of Food and Nutrition and the National Centre for Nutrition Education, and does not distinguish the intake of individual food types from each food category. However, this was done deliberately to avoid any negative impact on the length and content of this questionnaire. Finally, this survey reviews eating behaviour over a 10 years period, and the 2022 survey was conducted after the lockdown period associated with the COVID-19 pandemic, which may also distort the survey results. The study has the advantage of group size and the re-use of the same questionnaire tool, so diet can be accurately compared over the years among the women surveyed.

## 5. Conclusion

Based on the study, we can draw the following conclusions: there have been changes in the diet of women living in Silesia (Poland) over the last decade. Disturbing trends were observed in the consumption of milk and dairy products, fast-food, salty snacks (chips, crisps) and sweets, which may contribute to the occurrence of diseases resulting from poor nutrition such as cardiovascular diseases, cancer, diabetes. At the same time, health-promoting dietary trends were observed in the study group of women, such as increased consumption of whole-grain cereals, vegetables, fruits and legumes. The results of the study will help determine the future direction of nutrition education, taking into account current dietary mistakes, in order to minimise the negative impact of nutrition on women’s future health and to formulate ministerial prevention programmes.

## Data availability statement

The raw data supporting the conclusions of this article will be made available by the authors, without undue reservation.

## Ethics statement

The studies involving human participants were reviewed and approved by the Bioethics Committee of the Silesian Medical University in Katowice in accordance with Resolution No. KNW/0022/KB1/151/I/11 of 06.12.2011. The patients/participants provided their written informed consent to participate in this study.

## Author contributions

AB-D and TK: conceptualization, methodology, and validation. AB-D and EC: formal analysis and investigation. AK and BC: resources. OS and AS-C: data curation. AB-D, MS-K, and WS: writing—original draft preparation. AB-D, EC, and WS: writing—review and editing. MK and MG: visualization. MM-W and ES: supervision. AB-D: project administration. All authors contributed to the article and approved the submitted version.

## Conflict of interest

The authors declare that the research was conducted in the absence of any commercial or financial relationships that could be construed as a potential conflict of interest.

## Publisher’s note

All claims expressed in this article are solely those of the authors and do not necessarily represent those of their affiliated organizations, or those of the publisher, the editors and the reviewers. Any product that may be evaluated in this article, or claim that may be made by its manufacturer, is not guaranteed or endorsed by the publisher.
